# Toward standardized geriatric-neurosurgical co-management: the transnational GENEUS project

**DOI:** 10.1007/s41999-026-01475-1

**Published:** 2026-04-21

**Authors:** Mariana Alves, Diogo Roque, Sofia Duque, Gabor Abellan van Kan, Nicolas Martinez Velilla, Eric Schmidt

**Affiliations:** 1 Unidade de Ortogeriatria, ULS de Santa Maria, Lisbon, Portugal; 2 Faculdade de Medicina, Universidade de Lisboa, Av. Prof. Egas Moniz, 1649-028 Lisbon, Portugal; 3 Departament of Neurosurgery, ULS de Santa Maria, Lisbon, Portugal; 4 Hospital CUF Descobertas, Lisbon, Portugal; 5 Nova Medical School, Lisbon, Portugal; 6 IHU HealthAge, Toulouse, France; 7 Maintain Aging Research Team, CERPOP, Université de Toulouse, INSERM, Université Paul Sabatier, Toulouse, France; 8 Geriatrics Department, Navarrabiomed, Hospital Universitario de Navarra (HUN), Universidad Pública de Navarra (UPNA), IdiSNA, Pamplona, Spain; 9 CIBER of Frailty and Healthy Aging (CIBERFES), Instituto de Salud Carlos III, Madrid, Spain; 10 Department of Neurosurgery, Toulouse University Hospital, Toulouse, France

Dear Editor,

We read with interest the recent scoping review by Jesuyajolu et al. [1] regarding geriatric-neurosurgical co-management services. The authors provided a valuable synthesis of the current landscape, highlighting that while the integration of geriatricians into neurosurgical wards yields promising outcomes, the field remains limited by heterogeneous models and a lack of standardized, high-level evidence. Through our EU-funded GENEUS (GEriatric NEUroSurgery) consortium [2], we are actively contributing to the field by developing a standardized, transnational framework designed to address these limitations.

**Fig. 1:**
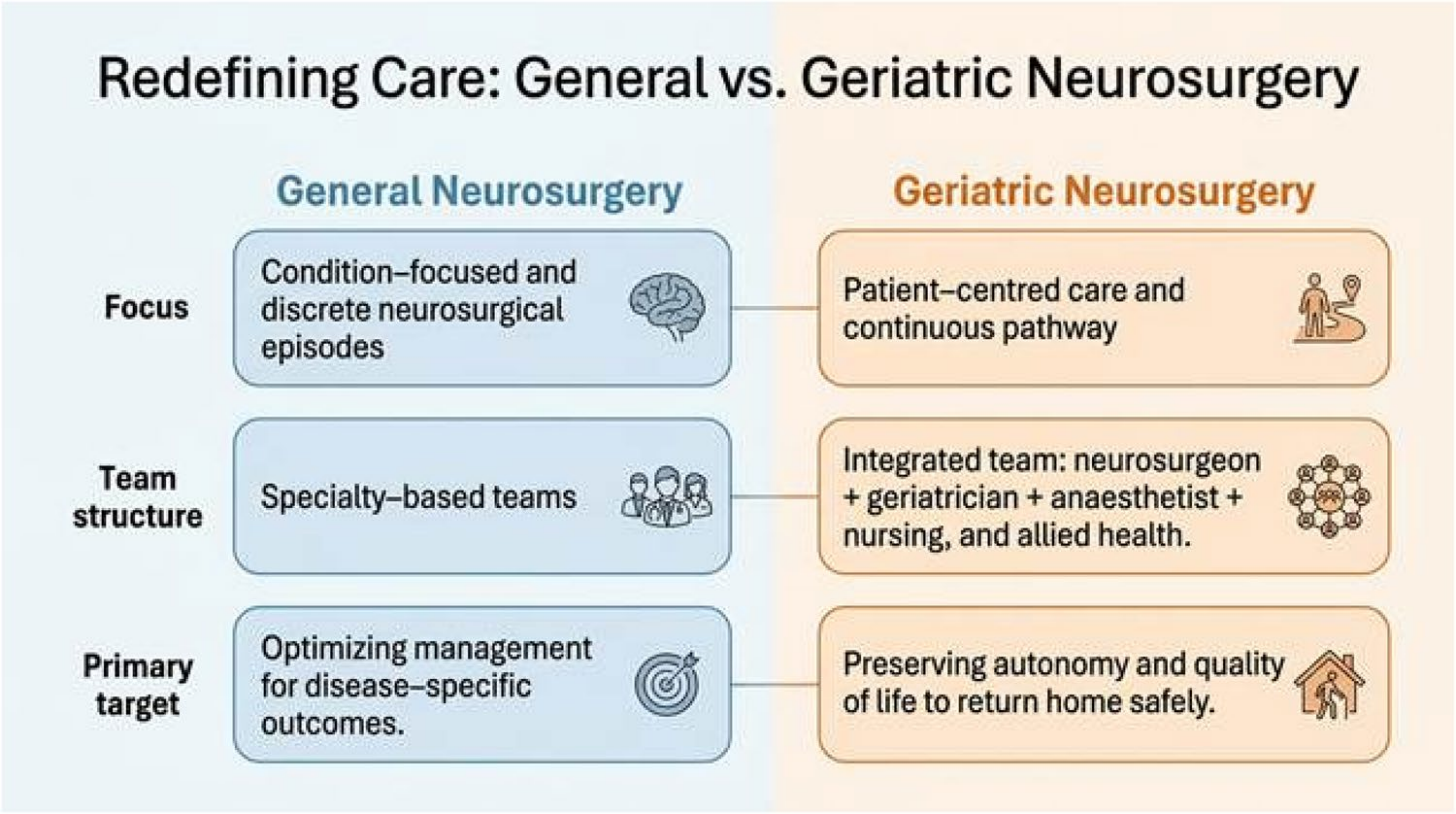
General vs. geriatric neurosurgery: from a disease-centered, episodic model to a patient-centered, multidisciplinary approach focused on functional outcomes and safe return home

To this heterogeneity, our group is working to harmonize care pathways across different health systems in Portugal, Spain, and France. Rather than introducing another isolated or additional local protocol, we are collaboratively designing an integrated care pathway for older neurosurgical patients. Specifically, the GENEUS project prospectively characterizes these patients and their trajectories, deliberately shifting the primary outcome from traditional measures of surgical success and overall survival to patient‑centered clinical and functional recovery (Fig. 1), with explicit attention to the modern geriatric concept of iatrogenic dependency.

Older neurosurgical patients are at risk of iatrogenic dependency**,** a loss of functional autonomy induced by healthcare systems rather than the underlying disease. Driven by factors such as long-lasting immobilization, excessive bladder catheterization or fluid administration, sedative exposure, and under-recognized delirium, this phenomenon can significantly affect long-term functional outcomes [3]. Drawing from the lessons learned in orthogeriatrics [4], the prevention of such complications supports the integration of structured geriatric assessment and targeted interventions to improve function [3].

This functional focus is supported by the development and implementation of a reproducible operational “toolbox,” allowing participating centers to systematically assess and manage key geriatric syndromes, such as delirium and functional decline. In parallel, the project will explore the cost-effectiveness of these integrated pathways within specific regional cohorts in France and in Spain.

We believe that cross-border data harmonization and multidimensional evaluation are necessary and the foundation for prospective, multicenter trials that the authors rightly call for.

Ultimately, facing an aging society, the reinforcement of comprehensive geriatric care within highly specialized surgical disciplines is no longer optional but a fundamental clinical imperative [5]. We commend the authors for their work, which reinforces the direction of our ongoing GENEUS European Interreg SUDOE initiative. We hope that our current efforts will soon provide robust evidence to support the widespread adoption of these co-management models.
